# Novel hematopoietic progenitor kinase 1 inhibitor KHK-6 enhances T-cell activation

**DOI:** 10.1371/journal.pone.0305261

**Published:** 2024-06-26

**Authors:** Min Jeong Ahn, Eun Hye Kim, Yunha Choi, Chong Hak Chae, Pilho Kim, Seong Hwan Kim

**Affiliations:** 1 Therapeutics & Biotechnology Division, Korea Research Institute of Chemical Technology, Daejeon, Republic of Korea; 2 Graduate School of New Drug Discovery and Development, Chungnam National University, Daejeon, Republic of Korea; 3 Medicinal Chemistry & Pharmacology, University of Science and Technology, Daejeon, Republic of Korea; Chittaranjan National Cancer Institute, INDIA

## Abstract

Inhibiting the functional role of negative regulators in immune cells is an effective approach for developing immunotherapies. The serine/threonine kinase hematopoietic progenitor kinase 1 (HPK1) involved in the T-cell receptor signaling pathway attenuates T-cell activation by inducing the degradation of SLP-76 through its phosphorylation at Ser-376, reducing the immune response. Interestingly, several studies have shown that the genetic ablation or pharmacological inhibition of HPK1 kinase activity improves the immune response to cancers by enhancing T-cell activation and cytokine production; therefore, HPK1 could be a promising druggable target for T-cell-based cancer immunotherapy. To increase the immune response against cancer cells, we designed and synthesized KHK-6 and evaluated its cellular activity to inhibit HPK1 and enhance T-cell activation. KHK-6 inhibited HPK1 kinase activity with an IC_50_ value of 20 nM and CD3/CD28-induced phosphorylation of SLP-76 at Ser-376 Moreover, KHK-6 significantly enhanced CD3/CD28-induced production of cytokines; proportion of CD4^+^ and CD8^+^ T cells that expressed CD69, CD25, and HLA-DR markers; and T-cell-mediated killing activity of SKOV3 and A549 cells. In conclusion, KHK-6 is a novel ATP-competitive HPK1 inhibitor that blocks the phosphorylation of HPK1 downstream of SLP-76, enhancing the functional activation of T cells. In summary, our study showed the usefulness of KHK-6 in the drug discovery for the HPK1-inhibiting immunotherapy.

## Introduction

The adaptive immune system, including CD4^+^ helper T cells and CD8^+^ cytotoxic T cells, plays a crucial role in fighting cancer [1]. Because T cells can recognize cancer cells and kill them directly or indirectly by communicating with other immune cells, immunotherapy to activate T cells has recently emerged as a powerful anticancer therapeutic strategy [2].

T-cell activation is primarily initiated by co-stimulating signals triggered by the engagement of the T-cell receptor (TCR)-CD3 complex and the subsequent engagement of co-stimulatory molecules, such as the CD28 receptor. The activation of intracellular signaling cascades mediates the release of cytokines and proliferation of T cells [3]. Interestingly, several TCR-activated intracellular molecules positively or negatively regulate the function of T cells [4]. Intracellular factors inhibiting TCR responses could be useful therapeutic targets to potentially enhance T-cell activity against cancers [5].

Hematopoietic progenitor kinase 1 (HPK1) is markedly expressed in a subset of hematopoietic cell lineages, and several studies have demonstrated that HPK1 functions as a negative regulator in TCR-activated signaling cascades [6]. TCR stimulation activates HPK1; subsequently, activated HPK1 phosphorylates the signaling scaffolding/adaptor protein SH2 domain-containing leukocyte protein of 76 kDa (SLP-76) at the serine 376 residue and mediates their ubiquitination and proteasomal degradation, leading to the attenuation of TCR signaling [7–10]. Interestingly, the genetic ablation or pharmacological inhibition of HPK1 kinase activity in cells and animal models have been shown to improve the immune response to cancers, suggesting that HPK1 could be a druggable target for T-cell-based cancer immunotherapy [10–14]. Novel HPK1 inhibitors have been discovered in academia and pharmaceutical companies; therefore, several HPK1 inhibitors are currently in clinical trials [15]; NDI-101150 of Nimbus Therapeutics and CFI-402411 of Treadwell Therapeutics are in phase 1/2 clinical trials for patients with solid cancer as monotherapy or in combination with the antiprogrammed cell death protein-1 (PD-1) monoclonal antibody pembrolizumab [16, 17], and PF-07265028 of Pfizer is in phase 1 clinical trials for patients with advanced or metastatic solid cancer as monotherapy or in combination with Sasanlimab to block the interaction between PD-1 and programmed cell death ligand-1 (PD-L1)/PD-L2 [18]. BGB-15025 of BeiGene is also in phase 1 clinical trials for patients with advanced solid cancer as monotherapy or in combination with the anti-PD-1 monoclonal antibody Tislelizumab [19]. By targeting the PD-1 pathway with a PD-1 inhibitor and HPK1 with an HPK1 inhibitor, the chance of broadening the immune response against cancer cells increases, potentially improving treatment outcomes. However, some tumors may have limited PD-L1 expression, making them less responsive to PD-1 inhibitors. Therefore, by combining an HPK1 inhibitor, which increases T-cell activation, with a PD-1 inhibitor, the immune response against the tumor could be further enhanced, even in cases where PD-L1 expression is low. This could be one of the reasons why the discovery and development of HPK1 inhibitors is actively underway [15]. In this study, we designed and synthesized a novel HPK1 lead compound, KHK-6, and evaluated its cellular activity to inhibit HPK1 and enhance T-cell activation.

## Materials and methods

### Synthesis of KHK-6

**Fig 1:**
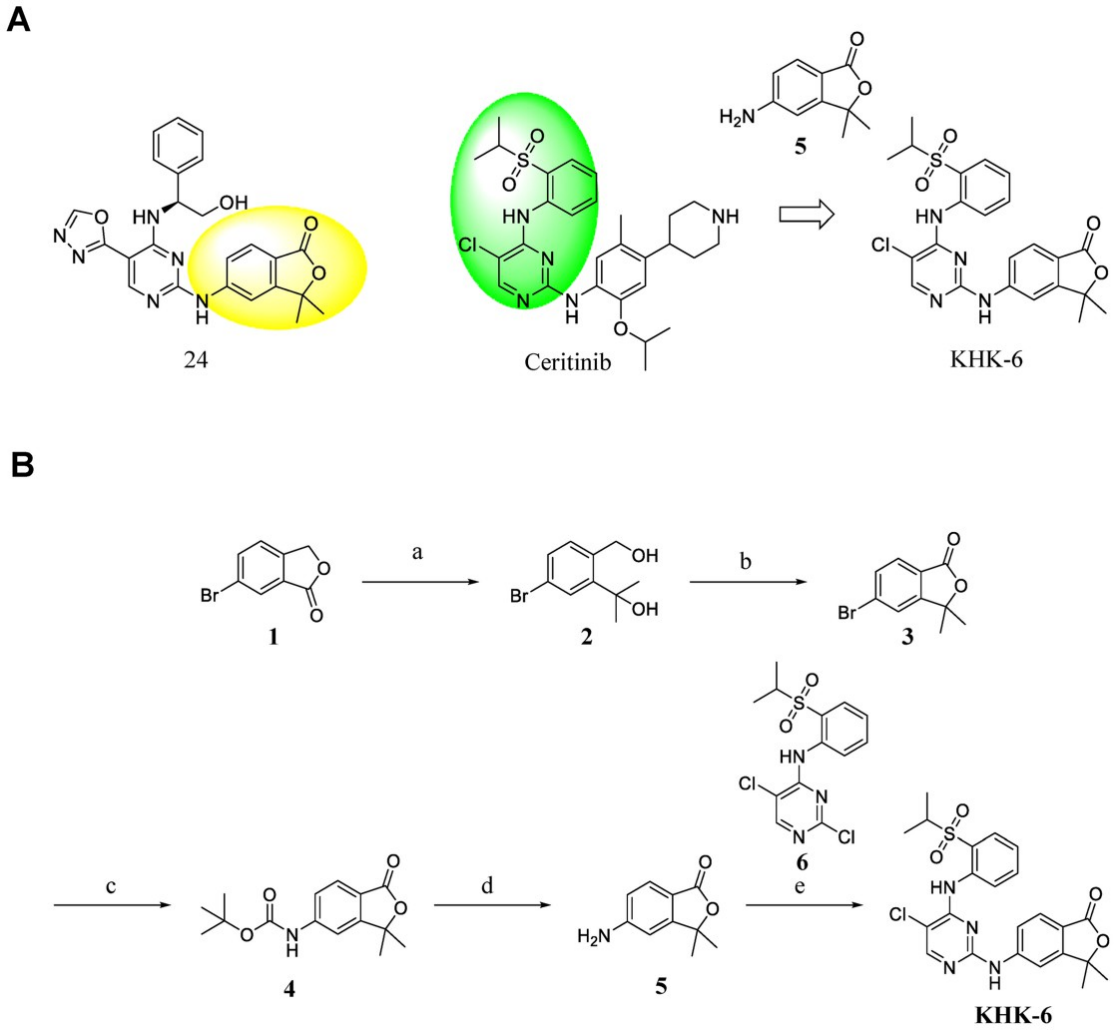
Design strategy and synthesis of KHK-6. (A) Design strategy for **KHK-6**. (B) Synthesis of **KHK-6**. Reagents and conditions: (a) 3 M CH_3_MgBr in Et_2_O, Et_2_O, 0 °C to rt, 3 h, 100%; (b) MnO_2_, THF, 70 °C, 16 h, 82%; (c) *ter*-butyl carbamate, Pd_2_(dba)_3_, Xantphos, Cs_2_CO_3_, 1,4-dioxane, 100 °C, 2 h, 44%; (d) 40% TFA in DCM, DCM, rt, 1 h, 81%; (e) PTSA·H_2_O, IPA, 90 °C, 16 h, 90%.

The synthesis of KHK-6 was launched from the preparation of amine **5**, as described in Fig 1 [20]. Dimethylation of commercially available 6-bromoisobenzofuran-1(3H)-one (**1**) was accomplished with methylmagnesium bromide to afford diol **2**. Oxidation of primary alcohol in 2 and subsequent cyclization produced lactone **3** in good yield using activated manganese dioxide [21]. To replace bromine **3** with the protected amine, Buchwald coupling with tert-butyl carbamate was performed to facilitate Boc-protected amine **4**. Deprotection of **4** was achieved under TFA conditions to deliver free amine **5**. Finally, the nucleophilic aromatic substitution of **5** with the commercially available pyrimidine **6** produced compound KHK-6. The detailed synthetic procedures are described in S1 File.

### Molecular modeling

Molecular docking studies were conducted using Maestro in Schrodinger Release 2023–3. The crystal structure of HPK1 (PDB code: 7KAC, https://www.rcsb.org/structure/7kac) was used with the structural defects fixed using the Protein Preparation Wizard module, and the ATP binding site of HPK1 was predicted using the crystal structure of HPK1 in complex with ATP analogue (AMPPNP; PDB code: 6CQD, https://www.rcsb.org/structure/6cqd) in RCSB PDB (Research Collaboratory for Structural Bioinformatics, The Protein Data Bank) [22]. The structure of KHK-6 was docked against the Adenosine triphosphate (ATP)-binding site using standard precision (SP)-flexible ligand docking protocols with SP options. The regeneration of the experimental conformation of crystal ligands with a good root–mean–square displacement of 0.2A shows the robustness of our docking protocols. The best docked poses with the lowest Glide score were selected for the subsequent binding pose analyses and molecular dynamics simulations. The molecular dynamics calculations were carried out using the Desmond program with default settings—SPC solvent model, orthorhombic periodic boundary conditions with neutralizing Na+ or Cl- ions, and NPT ensembles in 300 K and 1.01325 bar.

### HPK1 kinase activity assay

An HPK1 kinase activity assay was performed in Eurofins Discovery (St. Charles, MO, USA). Briefly, human recombinant HPK1 was incubated with MOPS (8 mM), ethylenediaminetetraacetic acid (0.2 mM), myelin basic protein (0.33 mg/mL), magnesium acetate (10 mM), and [γ-^33^P]-ATP (15 μM) at a pH of 7.0. The reaction was initiated by adding an Mg/ATP mixture. After incubation for 40 min at room temperature, the reaction was stopped by adding phosphoric acid to a concentration of 0.5%. Then, 10 μL of the reaction mixture was spotted onto a P30 filtermat and washed four times with 0.425% phosphoric acid and once with methanol for 4 min before drying and scintillation counting.

### Cell culture

The cells used in this study were obtained from Korean Cell Line Bank (Seoul, Korea), except for peripheral blood mononuclear cells (PBMCs) (ZenBio, Durham, NC, USA). Immortalized human T lymphocyte Jurkat cells and PBMCs were cultured in RPMI-1640 medium (HyClone, Logan, UT, USA) supplemented with 10% fetal bovine serum (FBS) (HyClone, Logan, UT, USA) and 1% penicillin–streptomycin (Gibco; Thermo Fisher Scientific, Waltham, MA, USA). Human ovarian cancer SKOV3 cells were cultured in McCoy’s 5a medium (HyClone, Logan, UT, USA) supplemented with 10% FBS and 1% penicillin–streptomycin. Human lung cancer A549 cells were cultured in Dulbecco’s Modified Eagle Medium (HyClone, Logan, UT, USA supplemented with 10% FBS and 1% penicillin–streptomycin.

### Cell viability assay

Jurkat cells and PBMCs (2 × 10^5^/well) in a 96-well plate were treated with KHK-6 and incubated for 3 days. Cell viability was then evaluated using the Cell Counting Kit-8 (CCK-8) assay (LPS solution, Seoul, Korea). Absorbance was measured using a Hidex sense microplate reader (Hidex, Turku, Finland) at 450 nm. The data were verified with at least three independent experiments.

### T-cell activation

Jurkat cells (2 × 10^5^/well) were transferred into a 96-well plate immobilized with 2 μg/mL of antiCD3 antibody (BD Biosciences, San Jose, CA, USA) and stimulated with 4 μg/mL of soluble antiCD28 antibody (BD Biosciences, San Jose, CA, USA). PBMCs (2 × 10^5^/well) were stimulated with prewashed Dynabeads Human T-Activator CD3/CD28 (Thermo Fisher Scientific, Waltham, MA, USA) in a ratio of 1:1 (Dynabeads:T cells). Activated cells were then separated from the mixture of cells and Dynabeads using the DynaMag^™^-96 Bottom (Thermo Fisher Scientific, Waltham, MA, USA).

### Western blotting

Jurkat cells in a 96-well plate at a density 2 × 10^5^ cells/well were treated with KHK-6 for 1 h and then stimulated with anti-CD3/CD28 for 4 h. Then, the cells pooled from eight wells were centrifuged at 300 × g for 5 min, washed with cold 1 × PBS, centrifuged again, and lysed with radioimmunoprecipitation assay (RIPA) lysis buffer (TransLab, Daejeon, Korea) on ice for 40 min. After centrifugation at 16,200 × *g* for 20 min at 4°C, the amount of proteins in the supernatants of cell lysates was quantified using the Pierce BCA Protein Assay Kit (Thermo Fisher Scientific, Waltham, MA, USA). Proteins were resolved by 4%–15% gradient gel (Bio-Rad, Richmond, CA, USA) electrophoresis at 100 V for 80 min and transferred to polyvinylidene difluoride membranes (Bio-Rad, Richmond, CA, USA). The transferred membranes were blocked with 1× blocking solution (ATTO, Tokyo, Japan) at room temperature for 1 h and incubated with the primary antibody diluted at 1:1000 in 5% bovine serum albumin overnight at 4°C. Primary antibodies against p-SLP-76 (S376) and SLP-76 were purchased from Cell Signaling Technology (Danvers, MA, USA), and actin antibody was purchased from Santa Cruz Biotechnology (Dallas, TX, USA). After washing with Tris-buffered saline with 0.1% Tween 20 (TBST) three times, the membranes were probed with horseradish peroxidase (HRP)-conjugated secondary antibody (Cell Signaling Technology, Danvers, MA, USA) diluted at 1:5000 in TBST with 5% nonfat dry milk at room temperature for 2 h. After washing with TBST three times, the signal from HRP using ECL Western detection reagents (Millipore Corporation, Billerica, MA, USA) was measured using WSE-6100 LuminoGraph (ATTO, Tokyo, Japan). Intensities of the bands obtained from three independent experiments were analyzed using ATTO Densitometry Software CS Analyzer 4, and the relative SLP-76-normalized intensities of p-SLP-76 to the control were presented.

### p-SLP-76 (Ser376) enzyme-linked immunosorbent assay (ELISA) assay

Relative quantification of p-SLP-76 on PBMCs was analyzed using PathScan^®^ Phospho-SLP-76 (S376) Sandwich ELISA Kit (Cell Signaling Technology, Danvers, MA, USA) according to the manufacturer’s protocol. Briefly, PBMCs in a 96-well plate at a density 2 × 10^5^ cells/well treated with KHK-6 for 1 h were stimulated with Dynabeads Human T-Activator CD3/CD28 for 25 min. Then, the cell pooled from three wells were centrifuged at 300 ×*g* for 5 min, washed with cold 1× PBS, centrifuged again, and lysed with lysis buffer (Cell Signaling Technology, Danvers, MA, USA) with 1 mM phenylmethylsulfonyl fluoride (Cell Signaling Technology, Danvers, MA, USA) on ice for 30 min. After centrifugation at 16,200 ×*g* for 20 min at 4°C, the diluted cell lysate with the sample diluent solution was added to an ELISA microplate well and incubated for 2 h. The reconstituted detection antibody was then added to the washed plate of each well, incubated at 37°C for 1 h, and incubated with HRP-linked secondary antibody at 37°C for 30 min. Then, 3,3’,5,5’-tetramethylbenzidine liquid substrate was added to the washed plate and incubated at 37°C for 10 min. After adding the stop solution, the absorbance of the final reactants was measured at 450 nm using a Hidex sense microplate reader within 30 min.

### Human IL-2 ELISA

IL-2 secretion in Jurkat cells was analyzed using the Human IL-2 ELISA Kit (LABISKOMA, Seoul, Korea) according to the manufacturer’s protocol. Briefly, cells were treated with KHK-6 and stimulated with 2 μg/mL immobilized anti-CD3 antibody and 4 μg/mL soluble anti-CD28 antibody for 2 days. Then, the supernatants of the cells were harvested by centrifugation at 300 ×*g* for 5 min, and then, the IL-2 concentrations in the supernatants were calculated from a standard curve generated using the kit standard sample IL-2 (pg/mL).

### Cell and cytokine profiling assay in human T-cell activation

T-cell phenotypes and secreted cytokines in PBMCs were analyzed using Human T-Cell Activation Cell and Cytokine Profiling Kit (Sartorius, Göttingen, Germany) according to the manufacturer’s protocol. Briefly, PBMCs (2 × 10^5^ cells/well) were stimulated using Dynabeads for 2 days, as described above, and then, the cell–supernatant mixture from each well was transferred into new assay plates and incubated with a mixture of Human T-Cell Companion kit (Sartorius, Göttingen, Germany) capture beads to measure the protein levels of IL-2 and granulocyte-macrophage colony-stimulating factor (GM-CSF). After incubation for 1 h, the cell–supernatant mixture collected by centrifugation was incubated with a cytokine detection cocktail for 1 h, and the fluorescent antibody panel cocktail (for separate CD3^+^ T cells, CD3^−^ non-T cells, CD3^+^CD4^+^ T helper cells, and CD3^+^CD8^+^ T cytotoxic cells based on the three different T-cell activation markers: early CD69, late CD25, and even later HLA-DR markers) with cell membrane integrity dye (R/Red) was added to distinguish live immune cells from dead cells. After incubation for 1 h, the cell–supernatant mixture was washed and collected by centrifugation, and then, the cell and cytokine profiles in the final reactants were evaluated using the Intellicyt iQue Screener PLUS platform with VBR Lasers (Sartorius, Göttingen, Germany).

### Immune cell killing assay

As the effector cells, PBMCs in a 96-well plate at a density 2 × 10^5^/well were treated with KHK-6 for 1 h and stimulated with Dynabeads Human T-Activator CD3/CD28 for 1 day. Simultaneously, the target cells (SKOV3 and A549 cells) were labeled with IncuCyte Cytolight Rapid Green (Sartorius, Göttingen, Germany) according to the manufacturer’s protocol, seeded in a 96-well plate at a density 4 × 10^3^ cells/well, and cultured for 1 day. Then, the effector cells were added at a ratio of 1:5 (the target cells:the effector cells) in the target cell-cultured plate, and IncuCyte^®^ Annexin V Dye (Sartorius, Göttingen, Germany) was added directly to the co-culture. The co-culture was incubated in the IncuCyte S3 Live-Cell Analysis System (Sartorius, Göttingen, Germany), and the area (μm^2^/image) of double-positive target cells (Cytolight Rapid Green^+^/Annexin V red Dye^+^) was automatically quantified using IncuCyte Base Analysis Software 2022A (Sartorius, Göttingen, Germany) to measure cell death.

### Statistical analyses

Statistical analyses were generated by two-tailed t-tests, and the results are presented as means ± standard error of the mean using GraphPad Prism 5 (GraphPad, La Jolla, California, USA). *P*-values < 0.05 were used to denote statistical significance.

## Results

### Design and synthesis of the novel HPK1 inhibitor KHK-6

Amine 5 was used as a privileged fragment from a recent report on the HPK1 inhibitor 24. Regarding ceritinib, N1-pyrimidine and C2-amine of pyrimidine generate strong hydrogen binding ability with the mainchain atoms of Glu92 and Cys94 in the hinge region of HPK1. A hybrid of kinase inhibitors could be instrumental in the discovery of novel scaffolds [23, 24]. Thus, we opted to incorporate amine 5 into the 2-position of pyrimidine in the core scaffold of ceritinib (green part in Fig 1A). KHK-6 was then synthesized as described in Fig 1B. A modified literature method was adopted to prepare amine 5 at an overall yield of 29% [20]. Commercially available pyrimidine 6 was coupled with amine 5 to facilitate KHK-6 at a yield of 90%. The structure and purity of KHK-6 were unambiguously confirmed using 1H nuclear magnetic resonance (^1^H NMR; details in S2 File) and liquid chromatography/mass spectrometry (LC/MS).

### Molecular modeling of KHK-6

**Fig 2:**
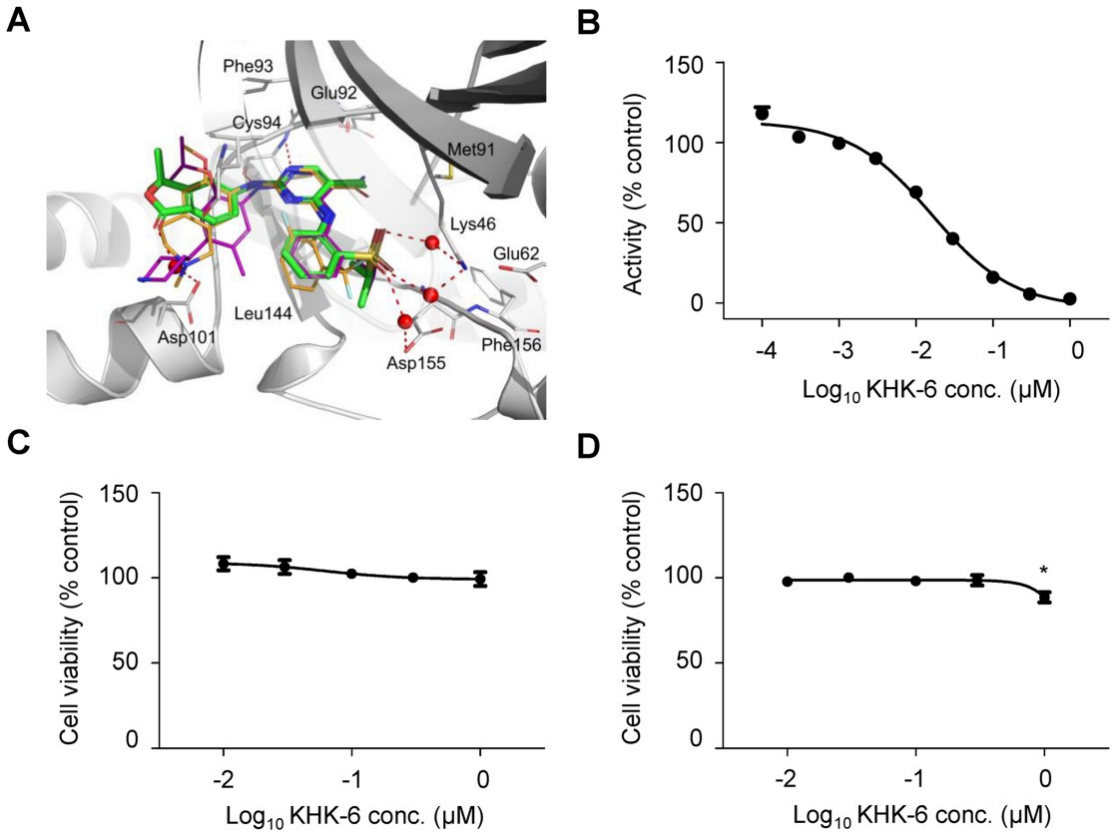
KHK-6 is a selective ATP-competitive HPK1 inhibitor. (A) Proposed binding mode of KHK-6 compound (green sticks) in ATP-binding site of HPK1 (gray ribbons) superimposed with the crystal structure of HPK1-IN-3 (orange sticks; in HPK1 crystal structure, 7M0M) and ceritinib (magenta sticks; in ALK crystal structure, 4MKC). Hydrogen bonding interactions are depicted in red dots, water molecules in red spheres, and flap regions of HPK1 are made transparent for clarity. Water positions were obtained by averaging the position of interacting water molecules in the trajectory of 250-ns molecular dynamics simulation of HPK1 and KHK-6 complex structure. (B) IC_50_ value was determined at a 1/3 dilution from 1 μM through KinaseProfiler^™^ by Eurofins. The data represent the mean values of two independent experiments. (C), (D) Cell viability was determined at a 1/3 dilution from 1 μM in Jurkat cells and PBMCs by the Cell Counting Kit-8 (CCK-8) assay. The data represent the mean values of three independent experiments. The data are presented as means ± standard error of the mean. * *P* < 0.05 (*vs*. negative control).

The potential of KHK-6 to inhibit the kinase activity of HPK1 could be explained by predicting their structural interpretation in a molecular modeling study (Fig 2A and S1 Fig). The binding affinity of KHK-6 was mainly ascribed to several direct or water-mediated H-bond interactions with HPK1. The two nitrogen atoms in the central aminopyrimidine ring form strong bidentate H-bonds with mainchain atoms of Cys94 in the hinge region. The sulfonyl forms two water-mediated H-bonds with the terminal nitrogen of Lys46 and the carboxylate group of Asp155. The carbonyl oxygen atom in benzofuranone also maintains water-mediated interactions with Asp101. Hydrophobic interactions of the central aminopyrimidine ring with Leu23, Val31, Ala44, and Leu144 also contribute to ligand binding. Overall, the proposed binding pose and ligand–protein interactions of KHK-6 compound were nearly identical to those of ceritinib and HPK1-IN-3 [25].

### KHK-6 inhibits HPK1 kinase activity

An *in vitro* HPK1 kinase activity assay revealed the potential of KHK-6 to inhibit HPK1 kinase activity with an IC_50_ value of 20 nM (Fig 2B). HPK1-IN-3 (S2A Fig) used as the reference compound exhibited an IC_50_ of 0.5 nM in an HPK1 kinase activity assay (S2B Fig).

### KHK-6 attenuated the induction of SLP76 phosphorylation during T-cell activation

**Fig 3:**
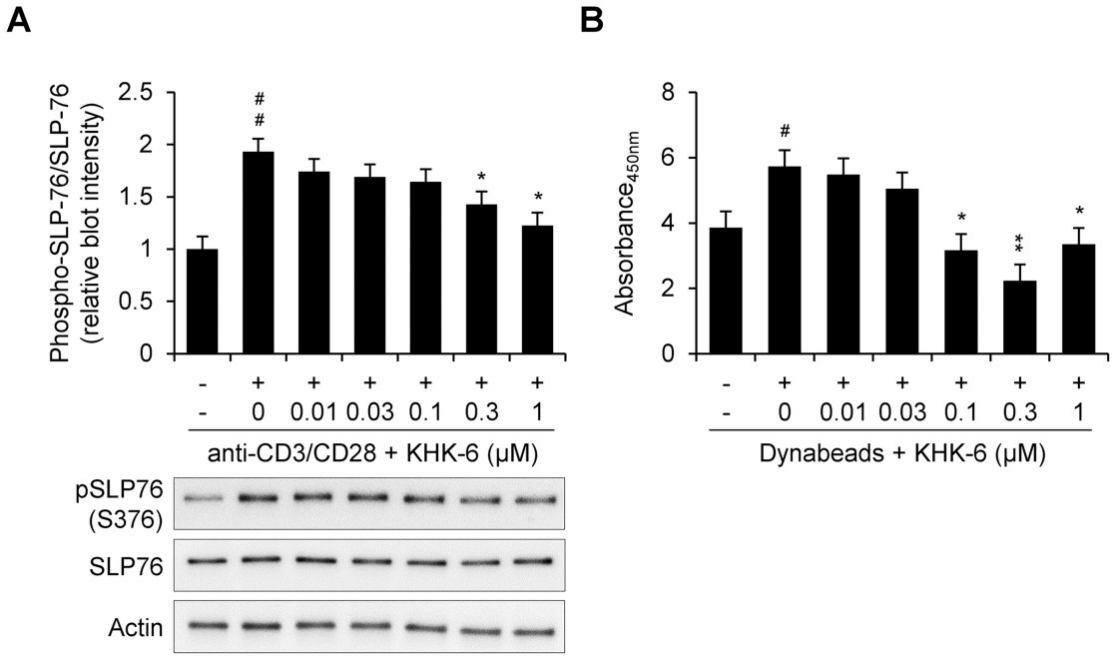
KHK-6 block serine376 phosphorylation of SLP-76 by TCR signaling. (A) Western blotting of Jurkat cells treated with each concentration of KHK-6 before stimulation for 1 h and stimulated with 2 μg/mL anti-CD3 (immobilized) and 4 μg/mL (soluble) anti-CD28 antibody for 4 h is shown. Relative blot intensity of p-SLP-76 (S376)/SLP-76 protein levels was analyzed. (B) Relative quantification of SLP76 protein phosphorylation on PBMCs treated with a specific concentration of KHK-6 before stimulation and stimulated with Dynabeads Human T-Activator CD3/CD28 at a ratio of 1:1 (Dynabeads: T cells) for 25 min was analyzed. The data are presented as means ± standard error of the mean. # *P* < 0.05; ## *P* < 0.01 (*vs*. negative control); * *P* < 0.05); ** *P* < 0.01 (*vs*. Dynabeads-stimulated control).

KHK-6 did not show any cytotoxic effect on Jurkat cells at the concentrations used in this study (Fig 2C). In PBMCs, the cytotoxicity of KHK-6 was observed at 1 μM, but not below 0.3 μM (Fig 2D). Then, to identify whether KHK-6 as an HPK1 inhibitor could inhibit the phosphorylation of the Ser376 residue in SLP76, the effect of KHK-6 on the protein levels of total SLP76 and p-SLP76 (S376) was evaluated in Jurkat cells. Western blotting revealed that KHK-6 dose-dependently inhibited the Dynabeads-induced phosphorylation of SLP-76 at serine 376 (Fig 3A); the anti-CD3/CD28-mediated activation of Jurkat cells significantly induced the relative expression of p-SLP-76 to SLP-76; however, its induction was dose-dependently inhibited by KHK-6 at 0.3 and 1 μM. Significant inhibition of SLP-76 phosphorylation by KHK-6 was also observed in PBMCs using a p-SLP-76 ELISA (Fig 3B); Dynabeads-mediated T-cell activation in PBMCs significantly induced the phosphorylation of SLP-76; however, KHK-6 significantly inhibited its induction at concentrations ranging from 0.1 to 1 μM.

### KHK-6 enhances the production of cytokines related to T-cell activation

**Fig 4:**
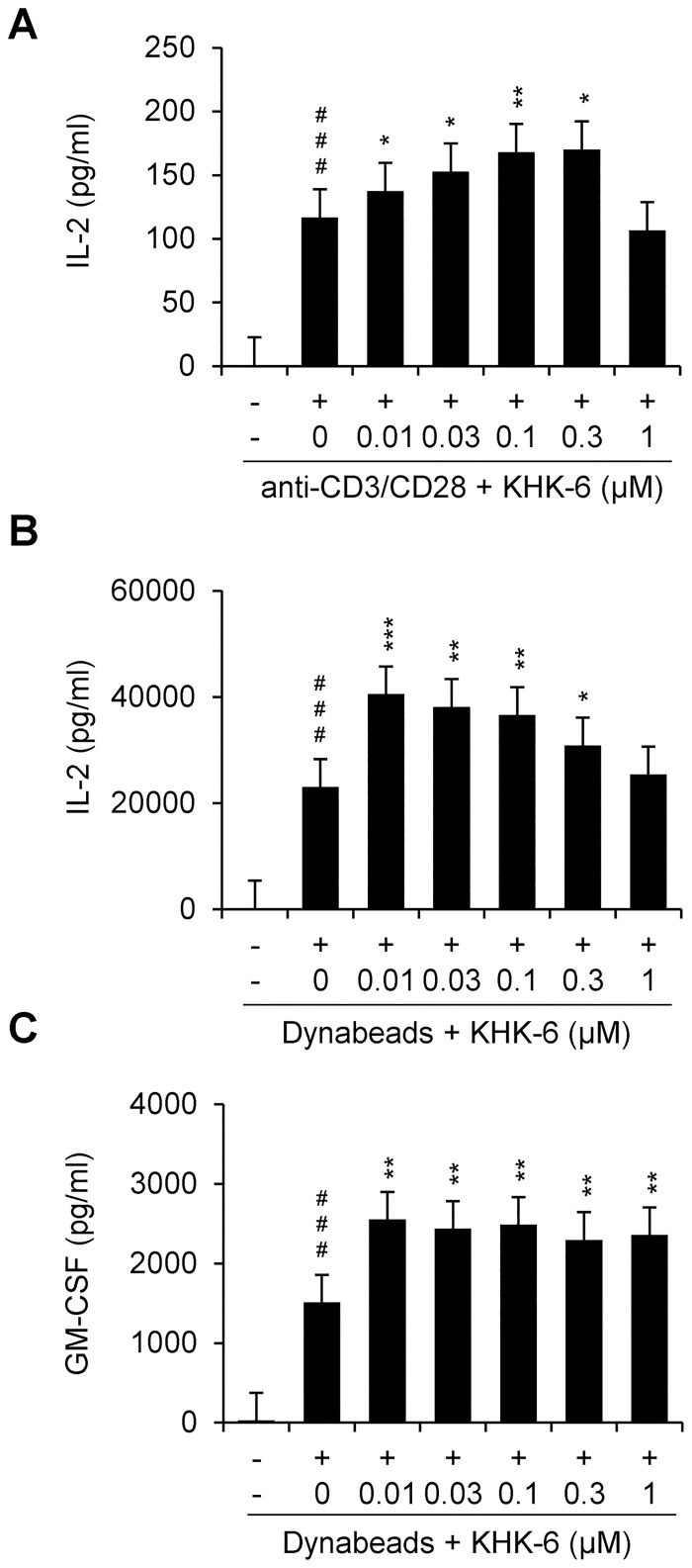
KHK-6 effects on cytokine release in activated T cells. (A) IL-2 production in Jurkat cells treated with a specific concentration of KHK-6 before stimulation and stimulated with 2 μg/mL (immobilized) anti-CD3 antibody and 4 μg/mL (soluble) anti-CD28 antibody for 2 day was analyzed using a human IL-2 ELISA. The data shown represent the results of three independent experiments. (B), (C) Cytokine production (IL-2 and GM-CSF) in PBMCs treated with specific concentrations of KHK-6 before stimulation and stimulated with Dynabeads Human T-Activator CD3/CD28 at a ratio of 1:1 (Dynabeads:T cells) at the indicated stimulation time. All data were obtained from at least three independent experiments. The data are presented as means ± standard error of the mean. ### *P* < 0.001 (*vs*. negative control); * *P* < 0.05; ** *P* < 0.01 (*vs*. Dynabeads-stimulated control).

The cellular activity of KHK-6 as an HPK1 inhibitor was further investigated to measure the production of IL-2. KHK-6 significantly enhanced the anti-CD3/CD28-mediated induction of IL-2 production in Jurkat cells at doses up to 0.3 μM (Fig 4A), and this enhancing activity of KHK-6 was confirmed in PBMCs (Fig 4B). The enhancing effect of KHK-6 on T-cell activation in PBMCs was further confirmed by measuring the level of GM-CSF; KHK-6 significantly enhanced the Dynabeads-mediated induction of GM-CSF production (Fig 4C). Without any cytotoxicity (S2C Fig), HPK1-IN-3 significantly enhanced the anti-CD3/CD28-mediated induction of IL-2 in Jurkat cells (S3A Fig). In PBMCs, the cytotoxicity of HPK1-IN-3 was observed at a dose of 0.1 μM (S2D Fig); however, HPK1-IN-3 significantly enhanced the Dynabeads-mediated induction of IL-2 and GM-CSF production at concentrations ranging from 0.1 to 1 μM (S3B and S3C Fig).

### KHK-6 enhances T-cell phenotypes

**Fig 5:**
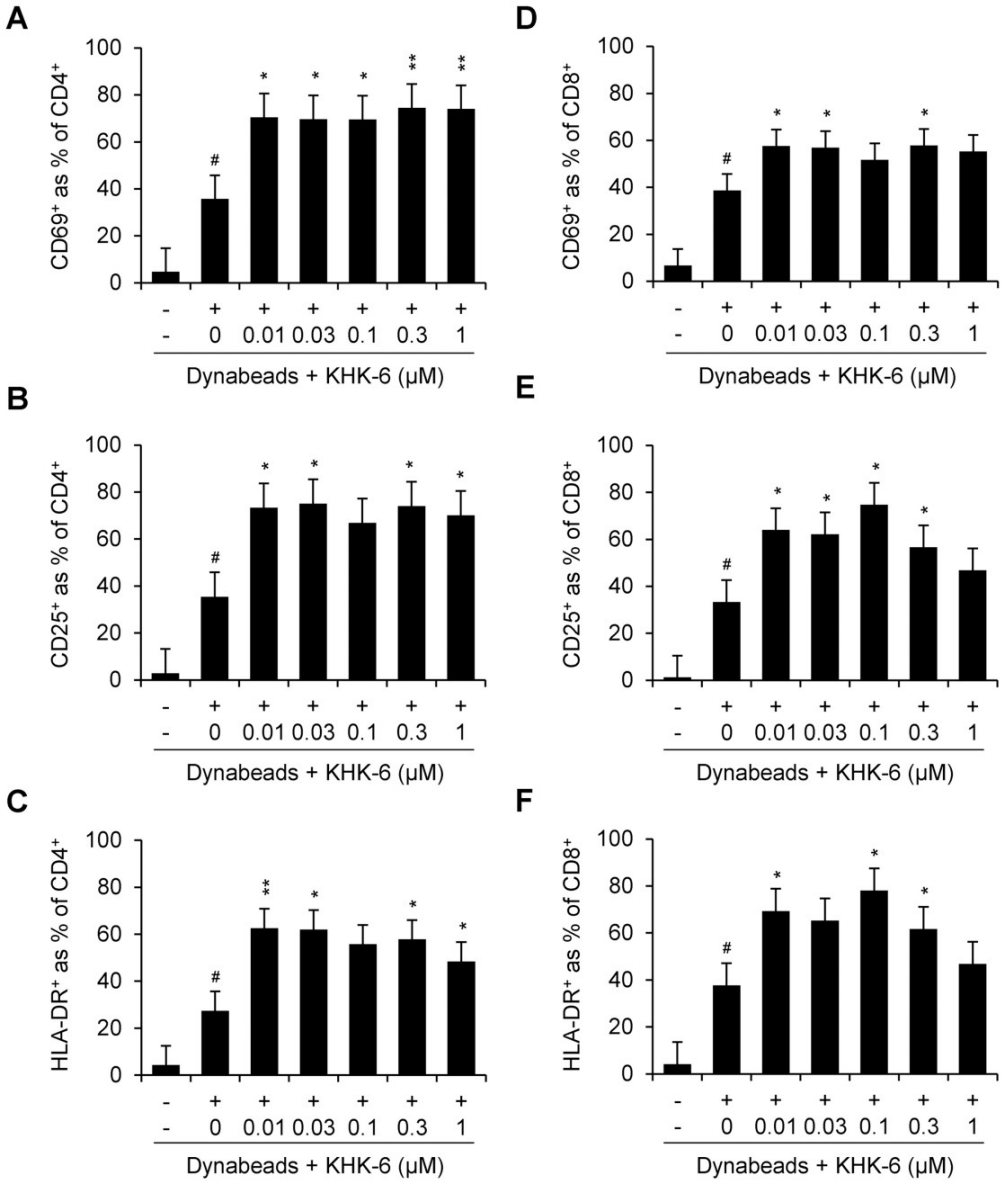
Effect of KHK-6 on CD4+ and CD8+ T-cell phenotypic marker expression. PBMCs were treated with KHK-6 for 1 h in the presence or absence of Dynabeads Human T-Activator CD3/CD28 stimulation. Different T-cell phenotypes of cells positive for early (CD69+) and late markers (CD25+ and HLA-DR+) among human CD4+T cells (A, B, and C) and CD8+ T cells (D, E, and F) were assessed using the iQue^®^ platform. All data were obtained from at least three independent experiments. The data are presented as means ± standard error of the mean. # *P* < 0.05 (*vs*. negative control); * *P* < 0.05; ** *P* < 0.01 (*vs*. Dynabeads-stimulated control).

The cellular activity of KHK-6 to enhance T-cell activation was investigated by evaluating changes in the proportion of CD4^+^ and CD8^+^ T cells expressing early (CD69), late (CD25), and even later (HLA-DR) T lymphocyte expression markers. Flow cytometric analysis revealed that the Dynabeads-mediated stimulation of PBMCs to T cells significantly increased all proportions of CD4^+^ and CD8^+^ T cells that express CD69, CD25, and HLA-DR markers, and this activity was significantly enhanced by KHK-6 (Fig 5). HPK1-IN-3 also significantly enhanced this activity, except for CD69^+^ CD8^+^ T cells (S4 Fig).

### KHK-6 enhances the tumor-killing activity of T cells

**Fig 6:**
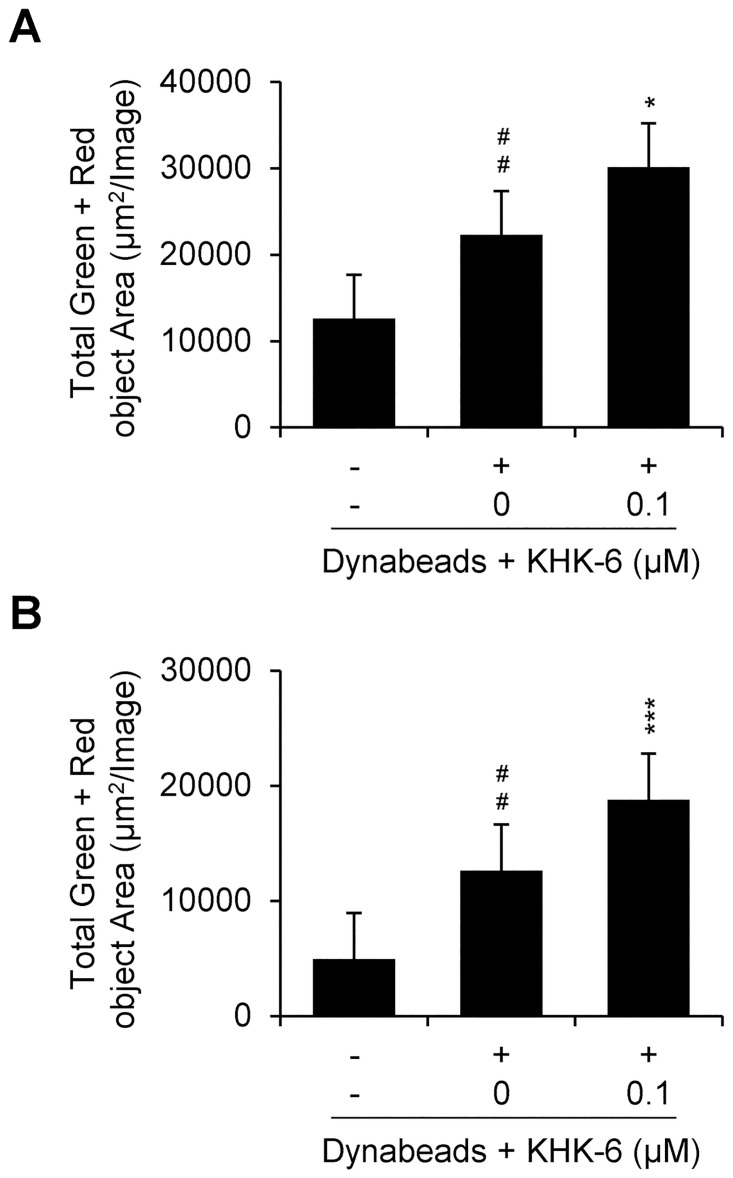
KHK-6 enhanced PBMC-mediated tumor-killing effect. Labeled SKOV3 (A) and A549 cells (B) co-cultured with PBMCs. Target cells (2 × 10^3^/well) were treated with KHK-6 before stimulation for 1 h and co-cultured with Dynabeads Human T-Activator CD3/CD28 stimulated effect cell. The ratio of effector cells (PBMCs) to the target cells (SKOV3 or A549) was 1:5. All data were obtained from at least three independent experiments. The data are presented as means ± standard error of the mean. ## *P* < 0.01; ### *P* < 0.001 (*vs*. negative control); * *P* < 0.05; *** *P* < 0.001 (*vs*. Dynabeads-stimulated control).

The intercellular activity of KHK-6 to enhance the tumor-killing activity of T cells was evaluated. The tumor-killing assay showed that the Dynabeads-mediated T-cell activation of PBMCs significantly increased the death areas of target cancer SKOV3 and A549 cells, and this activity was significantly enhanced by KHK-6 (Fig 6). The ability of HPK1-IN-3 to enhance the tumor-killing activity of T cells was also observed using SKOV3 cells (S5 Fig).

## Discussion

Enhancing the adaptive immune system to fight cancer, including B lymphocytes, CD4^+^ helper T lymphocytes, and CD8^+^ cytotoxic T lymphocytes, is a promising therapeutic strategy for cancer. HPK1 is a protein kinase that plays a crucial role in regulating various signaling pathways in immune cells, such as T and B cells. It acts as a negative regulator of TCR signaling, indicating that it dampens T cells activation. When HPK1 is active, it can inhibit T-cell function, reducing the immune response. Several studies have shown that HPK1 loss of function in mice enhanced T-cell activation and that HPK1 inhibitors are being developed with much interest, and some of them are in clinical trials [15].

In this study, we designed and synthesized KHK-6 and evaluated its biological activity related to T-cell activation. KHK-6 clearly inhibited the kinase activity of HPK1 and the phosphorylation of its downstream SLP-76 in the process of T-cell activation, indicating that KHK-6 can inhibit HPK1 and downregulate the HPK1-SLP-76 signaling cascade in the TCR signaling pathway. SLP-76 participates in TCR signaling and plays an important role in downstream signal transduction to activate various cellular responses. The protein activity of SLP-76 is regulated by phosphorylation, which influences its transmission of TCR signaling to different effector molecules [26]. It has been reported that HPK1 attenuates T-cell activation by inducing ubiquitination and proteasomal degradation of SLP-76 through its phosphorylation at the Ser-376 residue [10]. Notably, the structure-based discovery of diaminopyrimidine carboxamide as an HPK1 inhibitor showed to downregulate SLP-76 phosphorylation [25, 27, 28]. Since SLP76 has been shown to undergo its phosphorylation in a time-dependent manner [9, 29, 30], the further time kinetics experiments to completely conclude the effect of KHK-6 on the TCR-proximal signaling events would be required.

Activated T cells by the inhibition of HPK1-MAPK-NF-κB signaling cascades could produce and consume proliferative/survival cytokines [26, 31, 32] Among them, IL-2 and GM-CSF are involved in CD4^+^ and CD8^+^ T-cell-based anticancer efficacy. When T cells recognize cancer cells, various signaling cascades are initiated, including the MAPK signaling pathway involved in T-cell responses [3, 33]. IL-2 produced by activated T cells promotes the proliferation and differentiation of cytotoxic T lymphocytes as an autocrine mode or augments the primary and secondary CD8^+^ T-cell responses as a paracrine mode [34, 35]. Moreover, GM-CSF improves the maturation and function of dendritic cells, which are important in presenting antigens to CD8^+^ T cells and initiating their activation [36]. In this study, KHK-6 enhanced the TCR-induced release of IL-2 and GM-CSF and the activation of CD4^+^ and CD8^+^ T cells.

The ability of KHK-6 to enhance T-cell phenotypes could be a consequence of inhibiting the HPK1-mediated TCR signaling pathway, and HPK1 inhibition with the potential to enhance T-cell phenotypes indicated the enhanced immune cell activation, functionality, and tumor-specific immune responses. In particular, KHK-6 increased the population of CD8^+^ cytotoxic T cells, which are the most potent effectors in the anticancer immune response [37]. Upon recognition of tumor antigens, CD8^+^ T cells are activated and IL-2 can enhance the antitumor immune response mediated by CD8^+^ T cells. It promotes the expansion of activated CD8^+^ T-cell population, increasing their numbers and cytotoxic potential. Therefore, HPK1 inhibition increases the activity of CD8^+^ T cells, which are directly involved in killing cancer. The enhanced population of CD8^+^ cytotoxic T cells by KHK-6 could represent the functional shift toward a more active and responsive immune state to improve antitumor immunity and therapeutic outcomes in the context of cancer, and this function shift was verified with the results showing the tumor-killing activity of KHK-6. HPK1 could control two of the major arms of T cell’s function, TCR signaling and NF-kB circuit [38–40]. Given that HPK1 is not expressed in any major organs, it is less likely that an inhibitor of HPK1 kinase activity would cause any serious side effects [12, 41]. However, the interplay between TCR signaling and NF-kB circuit has also been relevant to the unwanted events such as leukemogenesis [41–43]. Therefore, the unexpected side effects originated from the inhibition of HPK1-TCR/NF-kB signaling axis should be thoroughly observed in the pre-clinical study of HPK1 inhibitors such as KHK-6.

Here, KHK-6-mediated HPK1 inhibition enhanced anti-CD3/CD28-induced T-cell activation at low concentrations, but not at high concentrations. The instances of bell-shaped dose responses to the immuno-oncologic therapeutics have been reported in several studies [44–47]. Compound 1 reported to HPK1 inhibitor exhibited the bell-shaped effects on anti-CD3/CD28-stimulated human CD4^+^ and CD8^+^ T cells [46]. Glucocorticoid-induced TNFR-related gene (GITR; TNFRSF18), a receptor belonging to the TNFR superfamily (TNFRSF), is activated by GITRL. GITR is expressed at low levels on resting responder T lymphocytes and is up-regulated in T regulatory cells (Treg cells) and in activated T cells, indicating that GITR is a co-stimulatory receptor and an important target for cancer immunotherapy [47]. GITR agonist IBI37G5 effectively activated effector T cells, but the functional analyses including human PBMC cytokine releases and human CD4^+^ T cell activation revealed its bell-shaped dose responses [48]. Additionally, the bell-shaped response was also induced by other TNFRSF agonist antibodies [48]. These bell-shaped concentration-response curves of small molecule inhibitors, hormones and antibodies could be explained by several issues such as solubility (aggregation and colloidal formulation) and the stoichiometry of the antibody-receptor complex [45, 48–51]. Considering the predicted water solubility of KHK-6 in in the website SwissADME (http://www.swissadme.ch/), the bell-shaped response to KHK-6 would not be due to its solubility or colloidal aggregation. More over-saturated inhibition of HPK1 by KHK-6 might lead to its competition and insufficient engagement with anti-CD3/CD28 and thereby exhibited the falling-off response from the best efficacy. This might be why the bell-shaped activity of KHK-6 occurred. Therefore, the bell-shaped response to drugs suggested the necessity of the dose-determination strategy for immune-oncologic therapeutics to predict antitumor efficacy [48]. The optimal efficacious dosing of immune-oncologic therapeutics guaranteeing the safety in the preclinical trial would be a crucial factor to increase the clinical development success rate [52, 53].

Additionally, in order to explain the bell-shaped response, the off-target activity of KHK-6 against other kinases could not be excluded. Indeed, it is a great challenge to discover highly selective kinase inhibitors due to the sequence similarity in the catalytic domain of MAP4K family kinases including HPK1 [54], and the selectivity against HPK1 could be improved in the process of lead optimization by replacing the specific chemical motif corresponding the difference in the gatekeeper residue of ATP binding pocket between HPK1 and other family kinases [55]. Comp K, the effective orally active HPK1 inhibitor developed by Bristol-Myers Squibb, improved the selectivity of all MAP4K family members through the lead optimization [25]. Similar to the binding mode of Comp K with HPK1, two nitrogen atoms in the central aminopyrimidine ring of KHK-6 form strong bidentate H-bonds with mainchain atoms of Cys94 in the hinge region, and the sulfonyl forms two water-mediated H-bonds with the terminal nitrogen of the catalytic residue Lys46 and the carboxylate group of Asp155 of the DFG loop region, one of the most clearly altered regions of HPK1 protein [56, 57]. The carbonyl oxygen atom in benzofuranone also maintains water-mediated interactions with Asp101. Considering the similar binding mode of KHK-6 compared to Comp K, the kinase selectivity of KHK-6 against HPK1 might be expected, but the extensive kinome screening study should be carried out after the optimization of KHK-6 to clarify the optimized compound’s activity against off-target kinases.

Finally, KHK-6 could be defined as the main compound that inhibits HPK1, and it could be developed via its *in vivo* efficacy study and lead optimization in a further study not only as monotherapy but also as a method for increasing the therapeutic effect by administering them in combination with other anticancer drugs, such as anti-PD-1 monoclonal antibody-checkpoint inhibitor. The combination of HPK1 inhibitors with PD-1 inhibitors could be beneficial to overcome tumor immune evasion [32, 40, 58, 59]. PD-1 inhibitors block the interaction between PD-1 on T cells and PD-L1 on cancer cells, thereby preventing the immune checkpoint and allowing T cells to attack tumor cells. However, some tumors may have limited PD-L1 expression, making them less responsive to PD-1 inhibitors. By combining an HPK1 inhibitor, which increases T-cell activation, with a PD-1 inhibitor, the immune response against tumor cells can be improved, even in cases where PD-L1 expression is low. Furthermore, targeting both the PD-1 pathway with a PD-1 inhibitor and HPK1 with an HPK1 inhibitor could broaden the immune response against cancer cells, potentially leading to better treatment outcomes because different immune checkpoint pathways may be involved in suppressing T-cell function in the tumor microenvironment. Therefore, combining HPK1 inhibitors with PD-1 inhibitors is a potential therapeutic strategy in cancer immunotherapy.

In summary, KHK-6 exhibited the ability to inhibit HPK1 and enhance the tumor-killing activity of HPK1 via T-cell activation. The optimization and development of KHK-6 would increase the clinical benefits of HPK1-inhibiting immunotherapy strategy as monotherapeutic agents and their combination with other anticancer drugs in a future.

## Supporting information

S1 File Synthetic procedures of KHK-6. (PDF)

S2 File Synthetic procedures of KHK-6: ^1^H NMR. (PDF)

S1 Fig HPK1-IN-3 is a potent and selective ATP-competitive HPK1 inhibitor. Proposed binding mode of KHK-6 compound (green sticks) in ATP-binding site of HPK1 (gray ribbons) superimposed with the crystal structure of HPK1-IN-3 (orange sticks; in HPK1 crystal structure, 7M0M) and ceritinib (magenta sticks; in ALK crystal structure, 4MKC). Hydrogen bonding interactions are depicted in red dots, water molecules in red spheres, and flap regions of HPK1 are made transparent for clarity. Water positions were obtained by averaging the position of interacting water molecules in the trajectory of 250-ns molecular dynamics simulation of HPK1 and KHK-6 complex structure. The views of the binding mode of KHK-6 from glucose pocket (down side; A) and solvent exposed region (left side; B) were represented.
(TIF)

S2 Fig HPK1-IN-3 is a potent and selective ATP-competitive HPK1 inhibitor. (A) Chemical structure of HPK1-IN-3. (B) The IC50 value was determined at a 1/3 dilution from 1 μM through KinaseProfiler^™^ by Eurofins. The data represent the mean values of two independent experiments. (C), (D) Cell viability was determined at a 1/3 dilution from 1 μM in Jurkat cells and PBMCs by the Cell Counting Kit-8 (CCK-8) assay. The data represent the mean values of three independent experiments. The data are presented as means ± standard error of the mean. * P < 0.05; ** P < 0.01 (vs. negative control).
(TIF)

S3 Fig HPK1-IN-3 effects on cytokine release in activated T cells. (A) IL-2 production in Jurkat cells treated with specific concentrations of HPK1-IN-3 before stimulation and stimulated with 2 μg/mL (immobilized) anti-CD3 antibody and 4 μg/mL (soluble) anti-CD28 antibody for 2 days was analyzed using the human IL-2 ELISA. (B), (C) Cytokine production (IL-2 and GM-CSF) in PBMCs treated with specific concentrations of KHK-6 before stimulation and stimulated with Dynabeads Human T-Activator CD3/CD28 at a ratio of 1:1 (Dynabeads:T cells) at the indicated stimulation time. All data were obtained from at least three independent experiments. The data are presented as means ± standard error of the mean. ### P < 0.001 (vs. negative control); ** P < 0.01; *** P < 0.001 (vs. Dynabeads-stimulated control).
(TIF)

S4 Fig HPK1-IN-3 decreased T lymphocyte activation. PBMCs were treated with HPK1-IN-3 for 1 h in the presence or absence of Dynabeads Human T-Activator CD3/CD28 stimulation. Different T-cell phenotypes of cells positive for early (CD69^+^) and late markers (CD25^+^ and HLA-DR^+^) among human CD4^+^ T cells (A, B, and C) and CD8^+^ T cells (D, E, and F) were assessed using the iQue^®^ platform. All data were obtained from at least three independent experiments. The data are presented as means ± standard error of the mean. # P < 0.05 (vs. negative control); * P < 0.05 (vs. Dynabeads-stimulated control).
(TIF)

S5 Fig HPK1-IN-3 suppressed cancer cell proliferation. (A) Labeled SKOV3 cells co-cultured with PBMCs. The target cells (SKOV3, 2 × 10^3^/well) were treated with KHK-6 before stimulation for 1 h and co-cultured with Dynabeads Human T-Activator CD3/CD28 stimulated effect cell (PBMCs, 1 × 10^4^/well). The ratio of effector cells (PBMCs) to the target cells (SKOV3) was 1:5. The data were obtained from at least three independent experiments. The data are presented as means ± standard error of the mean. ## P < 0.01 (vs. negative control); * P < 0.05 (vs. Dynabeads-stimulated control).
(TIF)
