# Supplementary material for: Novel hematopoietic progenitor kinase 1 inhibitor KHK-6 enhances T-cell activation
Source: PLoS One. 2024 Jun 26;19(6):e0305261. doi: 10.1371/journal.pone.0305261 (PMC11207149; doi:10.1371/journal.pone.0305261)
Supplement: S1 File — (PDF) [file pone.0305261.s001.pdf]

S1. Synthetic procedures of KHK-6.

Procedure for the preparation of 2-5.

2-(5-Bromo-2-(hydroxymethyl)phenyl)propan-2-ol (2)

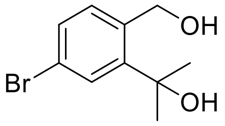

To a solution of 6-bromoisobenzofuran-1(3*H*)-one (**1**) (1.00 g, 4.69 mmol) in Et_2O (30 ml), nitrogen was charged for 5 min and then 3 M CH_3MgBr in Et_2O (4.69 ml, 14.1 mmol) was added dropwise at 0 °C for 20 min. The reaction mixture was stirred at rt for 3h. After the reaction was completed, the reaction mixture was cooled to 0 °C, and then quenched with sat. NH_4Cl (aq, 10 mL). The crude mixture was extracted with DCM, washed with brine, and dried with $MgSO_4$ to afford the desired compound (1.15 g, 4.69 mmol, 100%) as a colorless oil. ^1H NMR (500 MHz, $CDCl_3$) δ 7.45 (d, *J* = 2.1 Hz, 1H), 7.39 (dd, *J* = 8.1, 2.1 Hz, 1H), 7.21 (d, *J* = 8.1 Hz, 1H), 4.80 (s, 2H), 3.48 (s, 1H), 3.01 (s, 1H), 1.69 (d, *J* = 2.6 Hz, 6H).

5-Bromo-3,3-dimethylisobenzofuran-1(3H)-one (3)

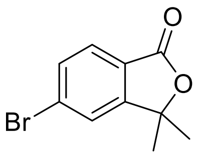

To a solution of **2** (1.15 g, 4.69 mmol) in THF (10 mL) was added 85% activated MnO_2 (4.08 g, 46.9 mmol) at rt. The reaction mixture was stirred at 70 °C for 16 h. After the reaction was completed, the reaction mixture was filtered with celite, and the residue was concentrated to afford the desired compound (926 mg, 3.84 mmol, 82%) as a white solid. ^1H NMR (400 MHz, $CDCl_3$) δ 7.75 (dd, *J* = 8.1, 0.6 Hz, 1H), 7.67 (dd, *J* = 8.1, 1.6 Hz, 1H), 7.59 (d, *J* = 1.6 Hz, 1H), 1.68 (s, 6H).

Tert-butyl (3,3-dimethyl-1-oxo-1,3-dihydroisobenzofuran-5-yl)carbamate (4)

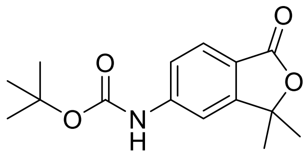

To an oven-dried sealed tube were added $Pd_2(dba)_3$ (57.0 mg, 0.0620 mmol) and Xantphos (36.0 mg, 0.0207 mmol) and then the mixture was placed under vacuum and refilled with nitrogen. To the sealed tube were added anhydrous 1,4-dioxane (2 mL), **3** (500 mg, 2.07 mmol), and *tert*-butyl carbamate (364 mg, 3.11 mmol). After Cs_2CO_3 (1.35 g, 4.15 mmol) was added, the mixture was stirred at 100 ºC for 2 h. After the reaction was completed, the reaction mixture was quenched with water, extracted with DCM, and concentrated under vacuum. The crude mixture was purified by silica gel column chromatography using 30% EtOAc in hexanes as eluent to afford the desired compound (250 mg, 0.901 mmol, 44%) as a white solid. ^{1}H NMR (400 MHz, DMSO-d_6) δ 9.73 (s, 1H), 7.92 (d, J = 2.0 Hz, 1H), 7.73 (dd, J = 8.3, 2.0 Hz, 1H), 7.60 (d, J = 8.3 Hz, 1H), 6.14 (s, 2H), 1.59 (s, 6H), 1.49 (s, 9H), 1.37 (s, 9H).

5-Amino-3,3-dimethylisobenzofuran-1(3*H*)-one (5)

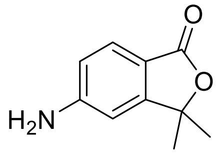

To a solution of **4** (250 mg, 0.901 mmol) was added 40% TFA in DCM (1.5 mL) and then stirred for 2 h. The reaction mixture was quenched with water, basified with sat. $NaHCO_3$ (aq), and extracted with DCM (30 mL x 2). The crude mixture was purified by silica gel column chromatography using 15% EtOAc in hexanes as eluent to afford the desired compound (130 mg, 0.901 mmol, 81%) a white solid. ^{1}H NMR (400 MHz, $CDCl_3$) δ 7.64 (d, J = 8.2 Hz, 1H), 6.71 (dd, J = 8.3, 2.0 Hz, 1H), 6.53 (d, J = 2.0 Hz, 1H), 4.30 (s, 2H), 1.62 (s, 6H).

Procedure for the preparation of 5-((5-chloro-4-((2-(isopropylsulfonyl)phenyl)amino)pyrimidin-2-yl)amino)-3,3-dimethylisobenzofuran-1(3*H*)-one (KHK-6)

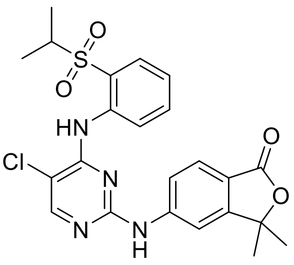

To a solution of 2,5-dichloro-*N*-(2-(isopropylsulfonyl)phenyl)pyrimidin-4-amine (**6**) (50.0 mg, 0.144 mmol) in IPA (1 mL) were added **5** (28.6 mg, 0.159 mmol) and PTSA·H_2O (29.8 mg, 0.173 mmol). The reaction mixture was stirred at 90 °C for 4 h. The reaction was quenched with water, basified with $NaHCO_3$ (aq), extracted with EtOAc, and washed with brine. The crude mixture was purified by silica gel column chromatography using 30% EtOAc in hexanes as eluent to afford the desired compound (68.9 mg, 0.141 mmol, 98%) as a white solid. ^{1}H NMR (400 MHz, DMSO-d_6) δ 10.15 (s, 1H), 9.43 (s, 1H), 8.45 (d, J = 8.2 Hz, 1H), 8.41 (s, 1H), 7.95–7.84 (m, 2H), 7.82 (t, J = 7.8 Hz, 1H), 7.73 (dd, J = 8.5, 1.8 Hz, 1H), 7.64 (d, J = 8.4 Hz, 1H), 7.46 (t, J = 7.7 Hz, 1H), 3.49 (s, 1H), 1.47 (s, 6H), 1.17 (s, 3H), 1.15 (s, 3H); LC/MS *m/z* 486.68 [M + H$^+$].
