# Supplementary material for: Novel hematopoietic progenitor kinase 1 inhibitor KHK-6 enhances T-cell activation
Source: PLoS One. 2024 Jun 26;19(6):e0305261. doi: 10.1371/journal.pone.0305261 (PMC11207149; doi:10.1371/journal.pone.0305261)

S2. Synthetic procedures of KHK-6 : ^{1}H NMR.

^{1}H NMR of 2-(5-bromo-2-(hydroxymethyl)phenyl)propan-2-ol (2)

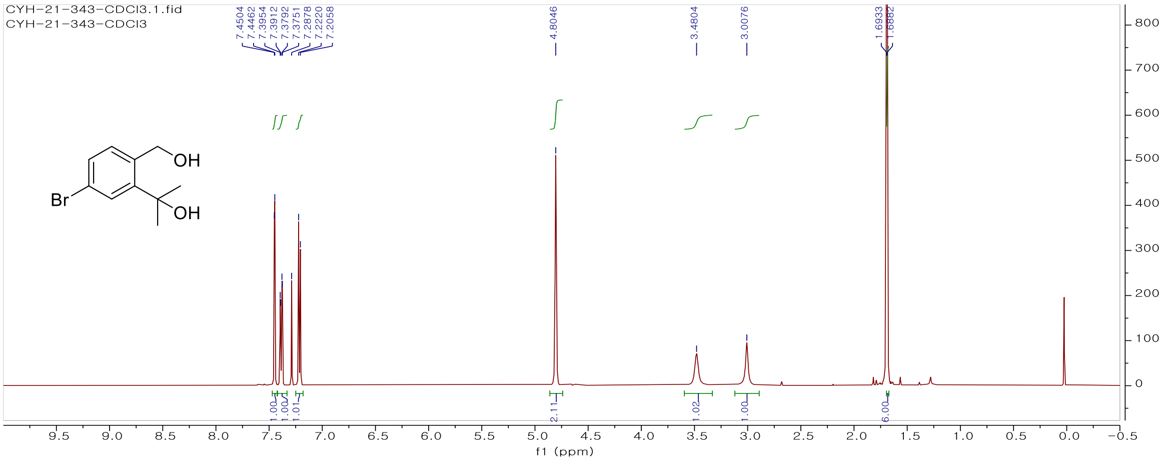

^{1}H NMR of 5-bromo-3,3-dimethylisobenzofuran-1(3H)-one (3)

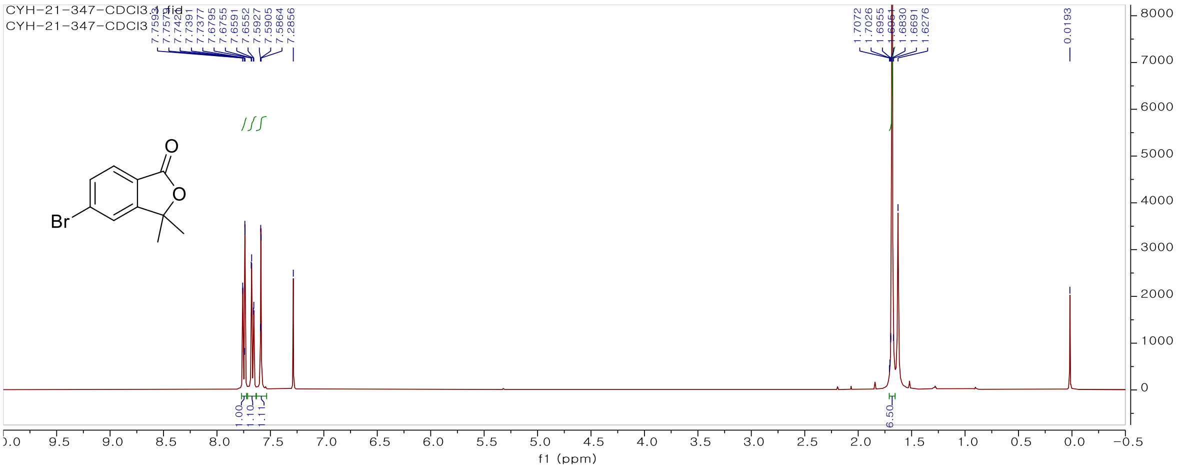

^{1}H NMR of *tert*-butyl (3,3-dimethyl-1-oxo-1,3-dihydroisobenzofuran-5-yl)carbamate (4)

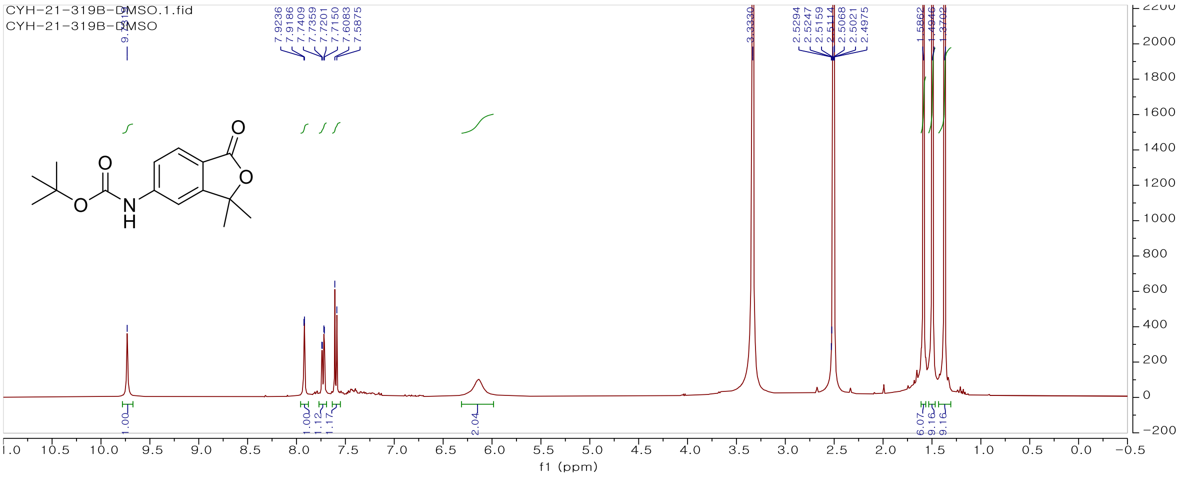

^{1}H NMR of 5-Amino-3,3-dimethylisobenzofuran-1(3*H*)-one (5)

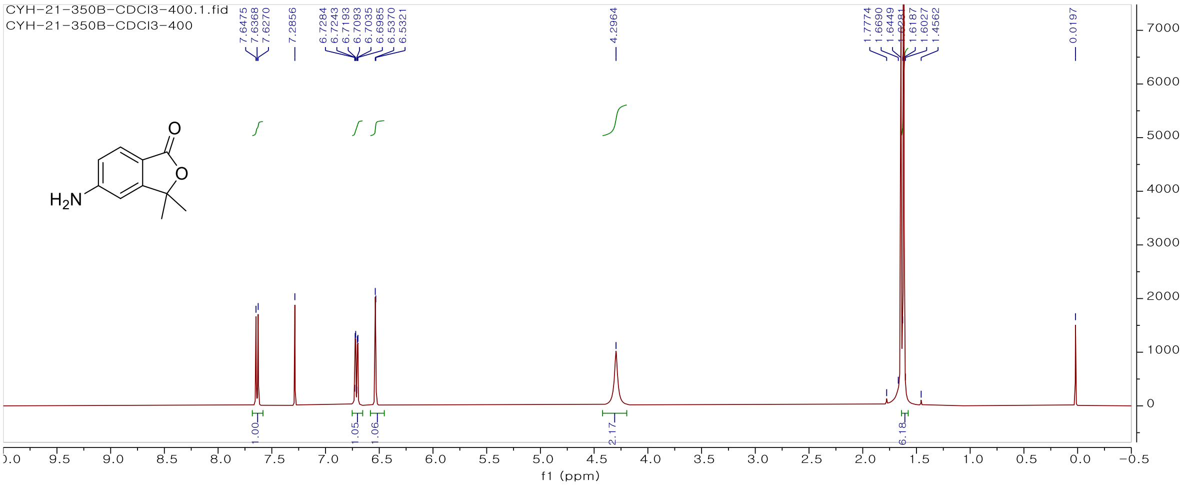

^{1}H NMR of 5-((5-Chloro-4-((2-(isopropylsulfonyl)phenyl)amino)pyrimidin-2-yl)amino)-3,3-dimethylisobenzofuran-1(3*H*)-one (KHK-6)

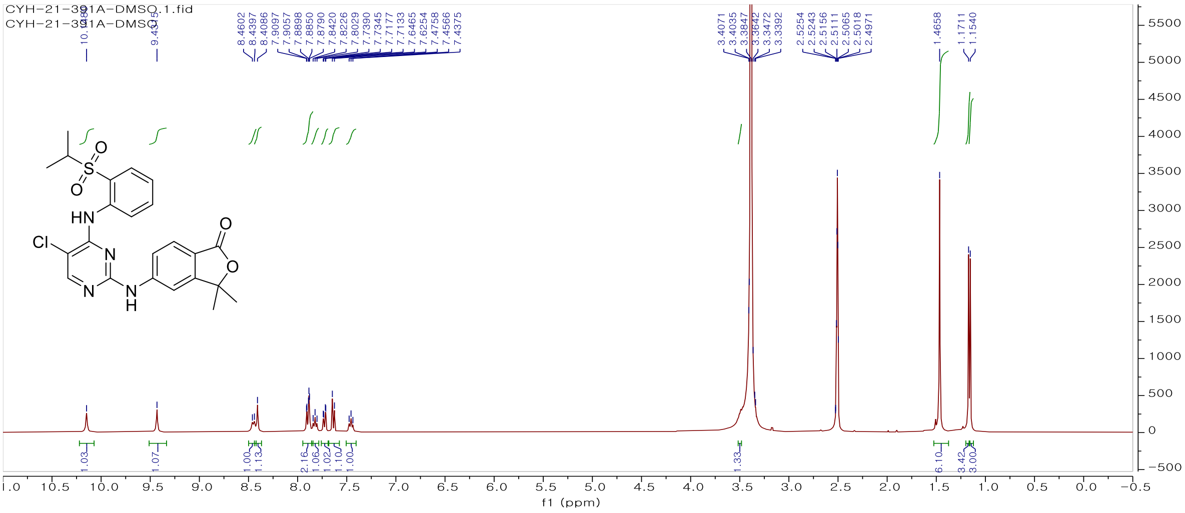

Supplement: S2 File — (PDF) [file pone.0305261.s002.pdf]
