# Supplementary figures and images for: Novel hematopoietic progenitor kinase 1 inhibitor KHK-6 enhances T-cell activation
Source: PLoS One. 2024 Jun 26;19(6):e0305261. doi: 10.1371/journal.pone.0305261 (PMC11207149; doi:10.1371/journal.pone.0305261)

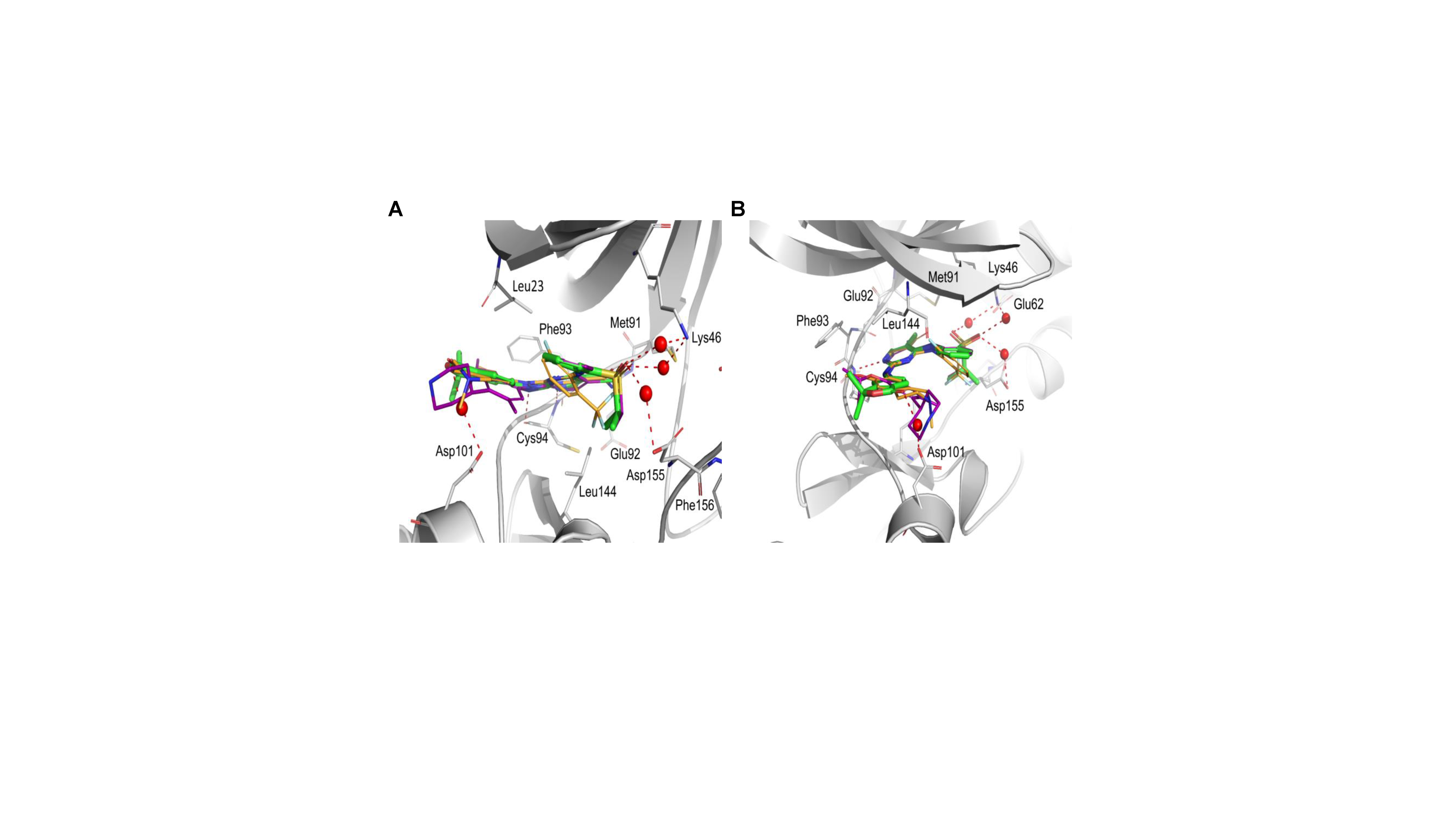

Supplement: S1 Fig — Proposed binding mode of KHK-6 compound (green sticks) in ATP-binding site of HPK1 (gray ribbons) superimposed with the crystal structure of HPK1-IN-3 (orange sticks; in HPK1 crystal structure, 7M0M) and ceritinib (magenta sticks; in ALK crystal structure, 4MKC). Hydrogen bonding interactions are depicted in red dots, water molecules in red spheres, and flap regions of HPK1 are made transparent for clarity. Water positions were obtained by averaging the position of interacting water molecules in the trajectory of 250-ns molecular dynamics simulation of HPK1 and KHK-6 complex structure. The views of the binding mode of KHK-6 from glucose pocket (down side; A) and solvent exposed region (left side; B) were represented. (TIF) [file pone.0305261.s003.tif]

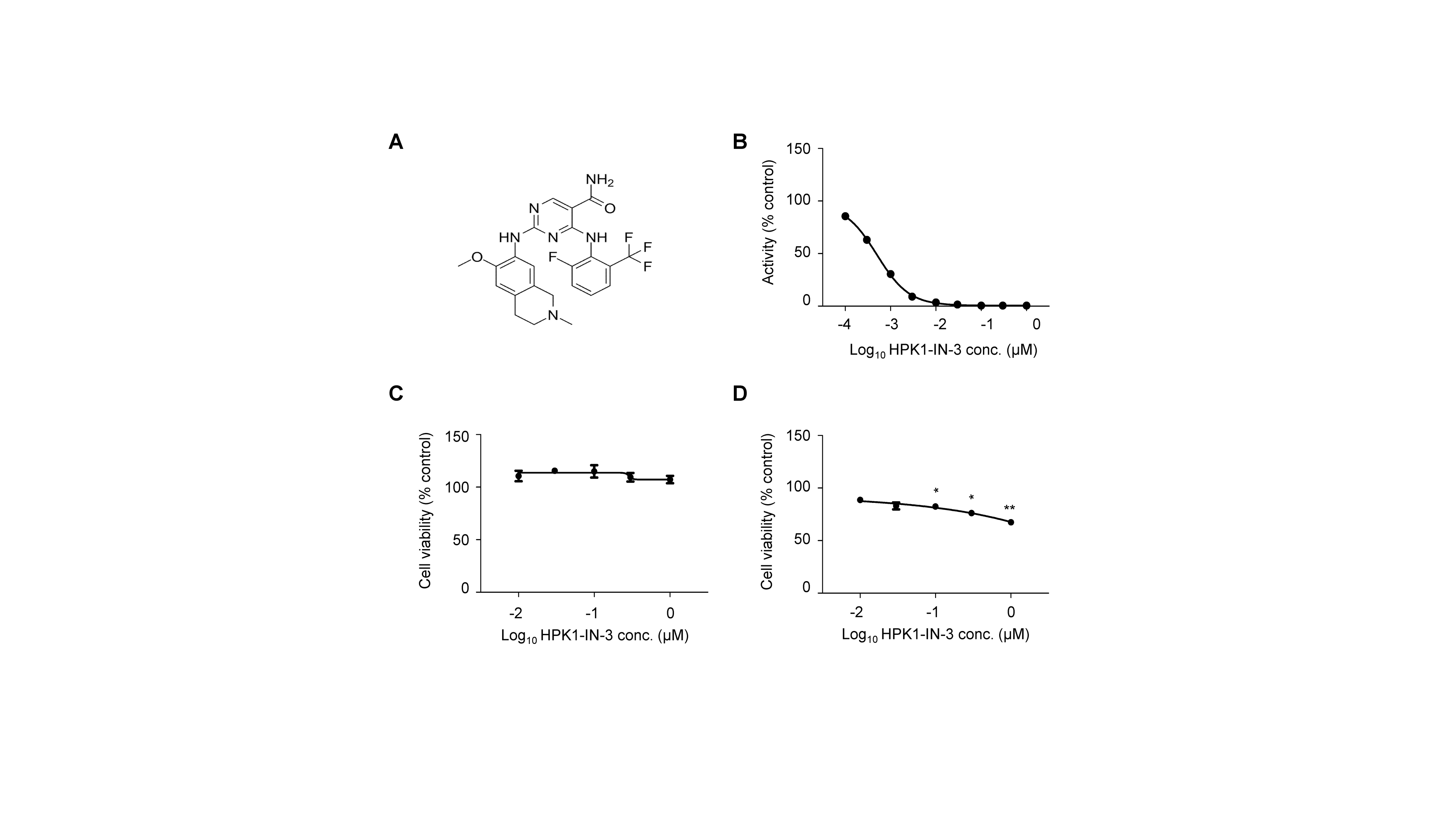

Supplement: S2 Fig — (A) Chemical structure of HPK1-IN-3. (B) The IC50 value was determined at a 1/3 dilution from 1 μM through KinaseProfiler™ by Eurofins. The data represent the mean values of two independent experiments. (C), (D) Cell viability was determined at a 1/3 dilution from 1 μM in Jurkat cells and PBMCs by the Cell Counting Kit-8 (CCK-8) assay. The data represent the mean values of three independent experiments. The data are presented as means ± standard error of the mean. * P < 0.05; ** P < 0.01 (vs. negative control). (TIF) [file pone.0305261.s004.tif]

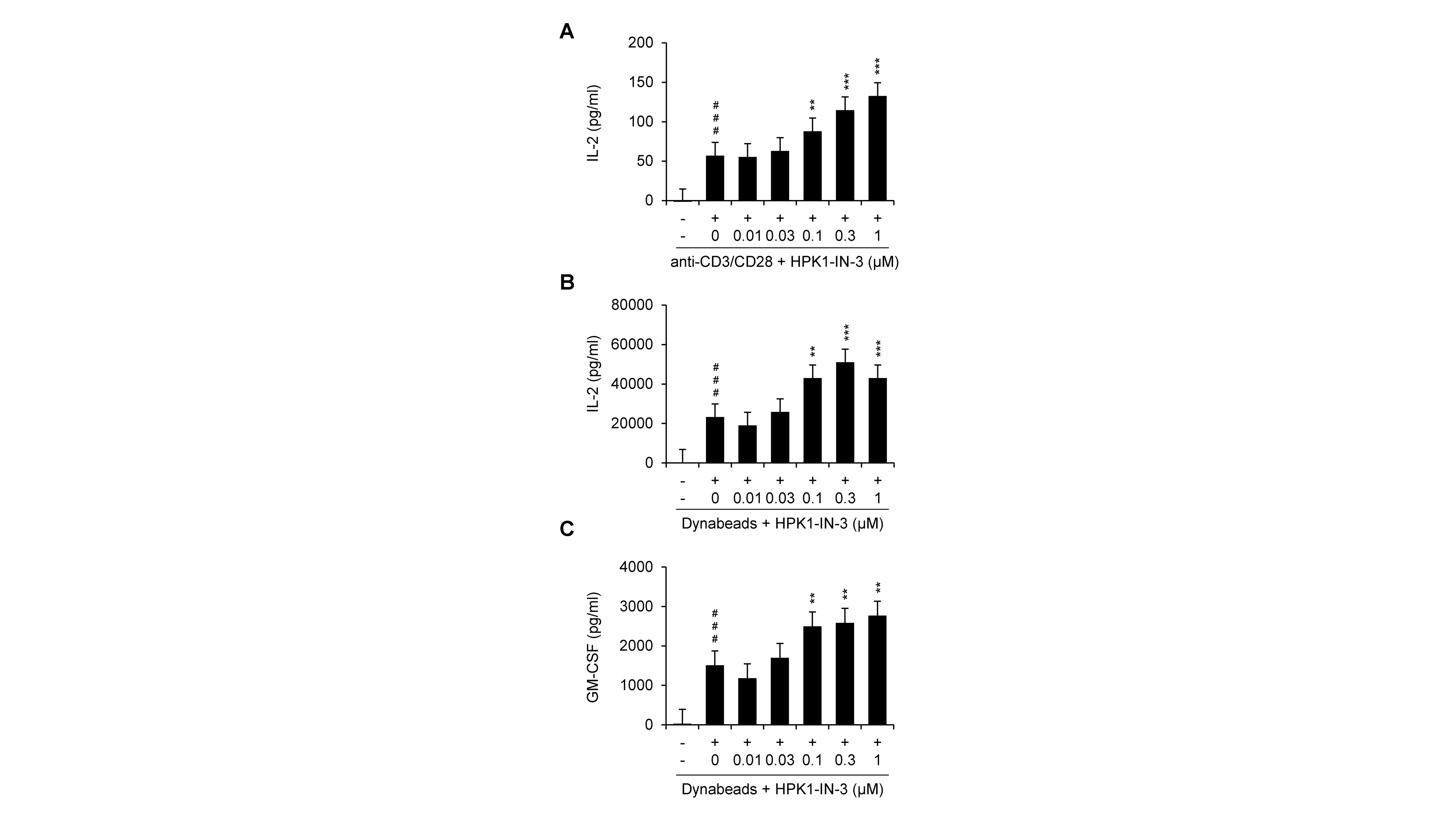

Supplement: S3 Fig — (A) IL-2 production in Jurkat cells treated with specific concentrations of HPK1-IN-3 before stimulation and stimulated with 2 μg/mL (immobilized) anti-CD3 antibody and 4 μg/mL (soluble) anti-CD28 antibody for 2 days was analyzed using the human IL-2 ELISA. (B), (C) Cytokine production (IL-2 and GM-CSF) in PBMCs treated with specific concentrations of KHK-6 before stimulation and stimulated with Dynabeads Human T-Activator CD3/CD28 at a ratio of 1:1 (Dynabeads:T cells) at the indicated stimulation time. All data were obtained from at least three independent experiments. The data are presented as means ± standard error of the mean. ### P < 0.001 (vs. negative control); ** P < 0.01; *** P < 0.001 (vs. Dynabeads-stimulated control). (TIF) [file pone.0305261.s005.tif]

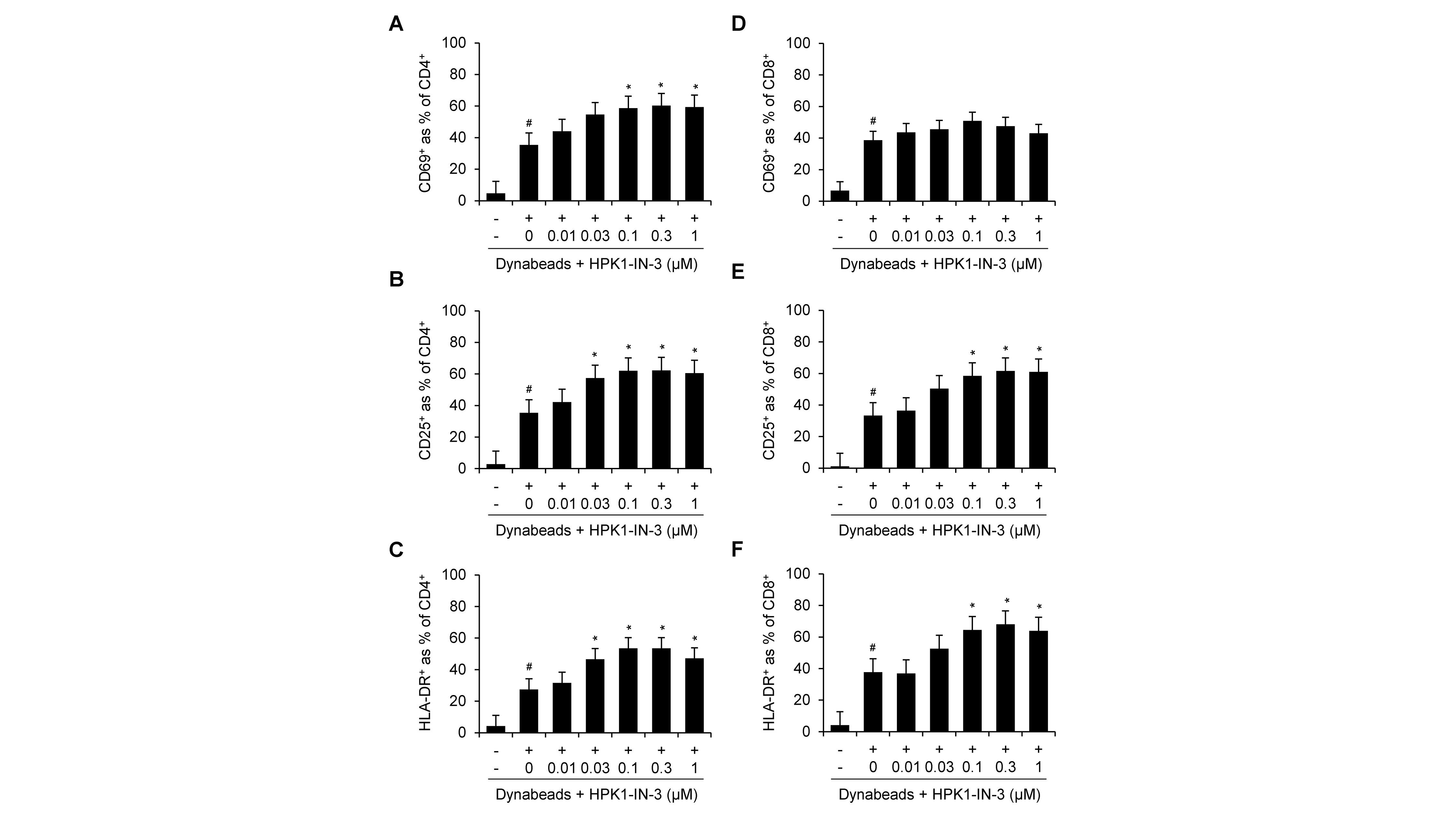

Supplement: S4 Fig — PBMCs were treated with HPK1-IN-3 for 1 h in the presence or absence of Dynabeads Human T-Activator CD3/CD28 stimulation. Different T-cell phenotypes of cells positive for early (CD69+) and late markers (CD25+ and HLA-DR+) among human CD4+ T cells (A, B, and C) and CD8+ T cells (D, E, and F) were assessed using the iQue® platform. All data were obtained from at least three independent experiments. The data are presented as means ± standard error of the mean. # P < 0.05 (vs. negative control); * P < 0.05 (vs. Dynabeads-stimulated control). (TIF) [file pone.0305261.s006.tif]

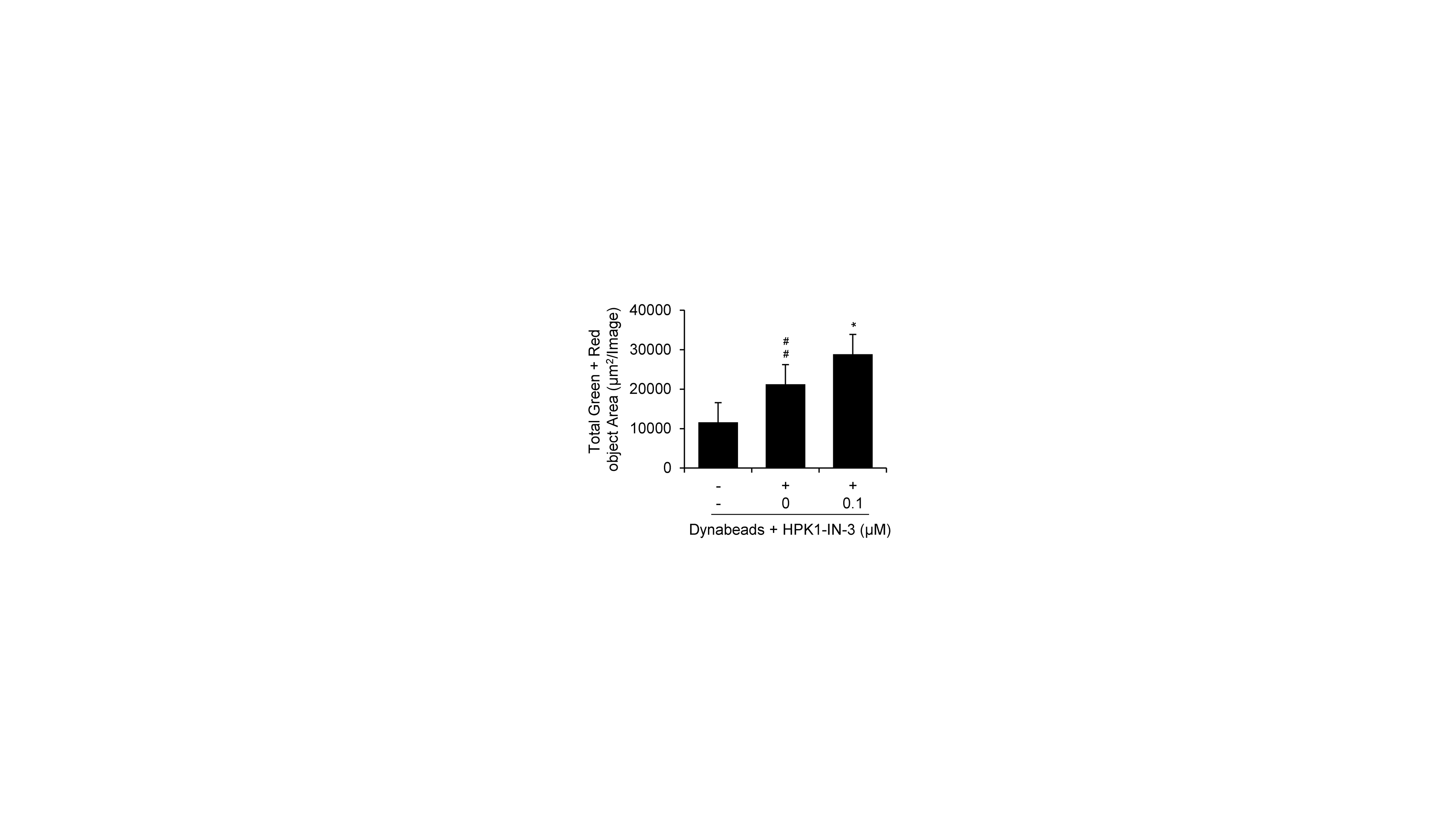

Supplement: S5 Fig — (A) Labeled SKOV3 cells co-cultured with PBMCs. The target cells (SKOV3, 2 × 10^3^/well) were treated with KHK-6 before stimulation for 1 h and co-cultured with Dynabeads Human T-Activator CD3/CD28 stimulated effect cell (PBMCs, 1 × 10^4^/well). The ratio of effector cells (PBMCs) to the target cells (SKOV3) was 1:5. The data were obtained from at least three independent experiments. The data are presented as means ± standard error of the mean. ## P < 0.01 (vs. negative control); * P < 0.05 (vs. Dynabeads-stimulated control). (TIF) [file pone.0305261.s007.tif]
